# Microbiota-Associated Metabolites as Biomarkers and Therapeutic Opportunities in Inflammatory Bowel Disease

**DOI:** 10.3390/nu18203372

**Published:** 2026-10-14

**Authors:** Krystian Krysa, Patrycja Musz, Gabriela Ryś, Paulina Serafin, Dawid Wachowiak, Sara Jarmakiewicz-Czaja

**Affiliations:** 1 Faculty of Health Sciences and Psychology, Collegium Medicum, University of Rzeszow, Warzywna 1a, 35-959 Rzeszow, Poland; pmusz@ur.edu.pl (P.M.); grys@ur.edu.pl (G.R.); pserafin@ur.edu.pl (P.S.); 2 University Center for Research and Development in Health Sciences, University of Rzeszow, Warzywna 1a, 35-959 Rzeszow, Poland; 3 Student Scientific Club of Human Nutrition, Faculty of Health Sciences and Psychology, Collegium Medicum, University of Rzeszow, Warzywna 1a, 35-959 Rzeszow, Poland; dw131242@stud.ur.edu.pl

**Keywords:** inflammatory bowel disease, gut microbiota, biomarkers, gut microbiota metabolites

## Abstract

Inflammatory bowel diseases, such as Crohn’s disease and ulcerative colitis, are chronic inflammatory conditions of the gastrointestinal tract characterized by periods of active disease and remission. Their pathogenesis is multifactorial and involves genetic predisposition, immune response, environmental factors, and alterations in gut microbiota composition and function. Growing evidence suggests that gut microbiota-associated metabolites not only reflect disease activity but also participate in regulating the immune response and maintaining the integrity of the intestinal barrier. The most important groups of metabolites associated with inflammatory bowel diseases include short-chain fatty acids, bile acids, and tryptophan derivatives. Reduced fecal butyrate and acetate concentrations have been reported in inflammatory bowel diseases, although the diagnostic value of fecal short-chain fatty acids remains uncertain. Potential therapeutic approaches include short-chain fatty acid supplementation, particularly sodium butyrate, and dietary interventions aimed at increasing short-chain fatty acid concentrations; however, the clinical efficacy of short-chain fatty acid supplementation remains inconclusive, and evidence for dietary interventions is limited. In the future, the use of gut microbiota metabolites may be based on personalized strategies grounded in multiomic data, enabling precise modulation of the microbiota–metabolite–immune system axis.

## 1. Introduction

**Figure 1:**
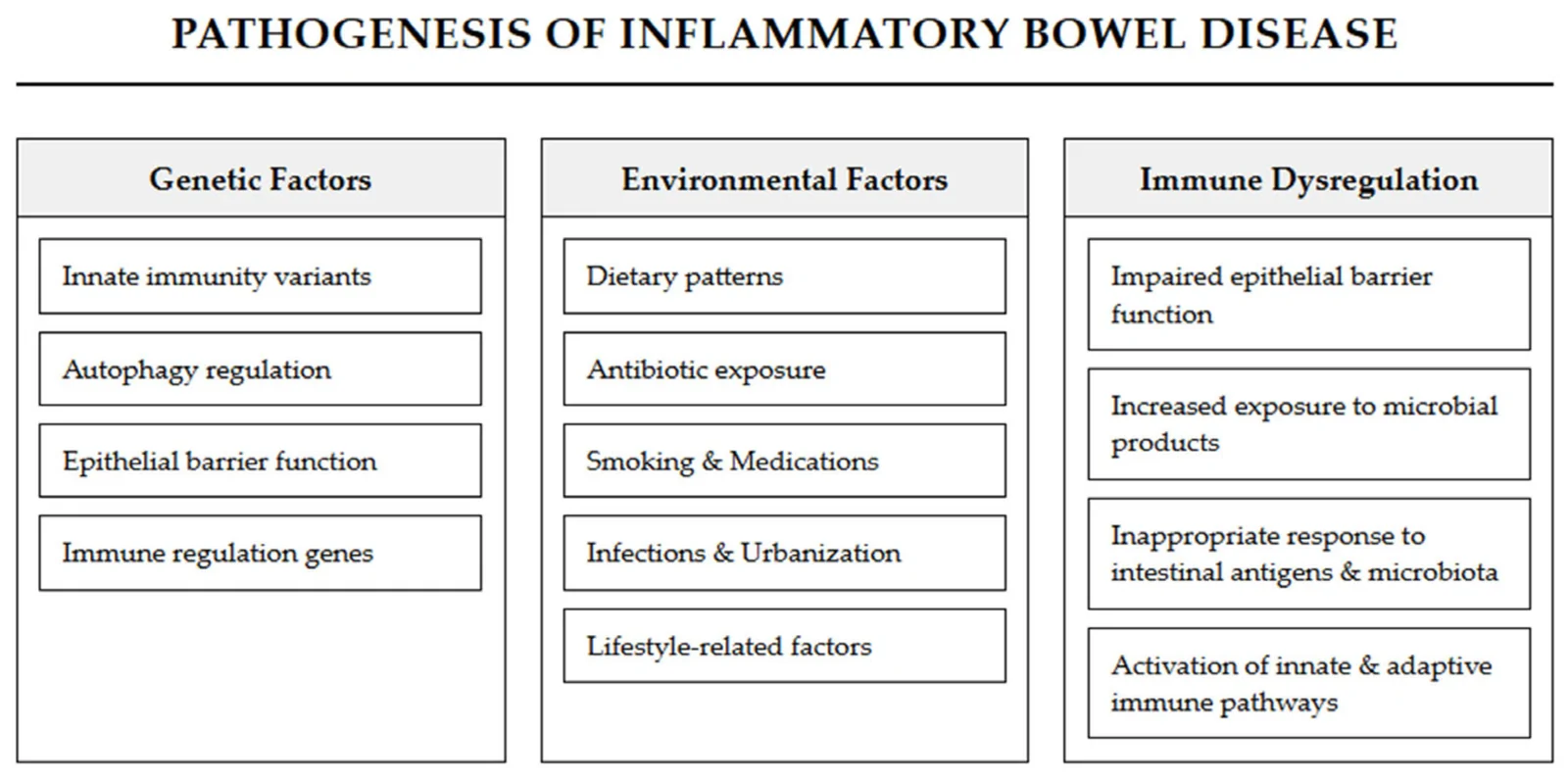
The pathogenesis of Inflammatory Bowel Disease (IBD).

Inflammatory bowel diseases (IBD) include Crohn’s disease (CD) and ulcerative colitis (UC). These are chronic conditions of the gastrointestinal tract with a complex etiology, characterized by periods of active phase and remission [1]. IBD prevalence rates vary by geographic region. Between 2000 and 2020, prevalence estimates in Europe ranged from 187 to 832 cases per 100,000 people, while in North America they ranged from 214.9 to 478.4 per 100,000 people [2]. Improvements in the economic status of the population and greater access to modern diagnostic methods have increased the detection rate of IBD, particularly in highly developed countries [1]. The pathogenesis of IBD involves environmental factors, abnormal regulation of the immune response, and genetic factors. Among the genetic factors, variants in genes involved in innate immunity, autophagy, epithelial barrier function, and immune regulation have been associated with IBD susceptibility. Environmental factors include dietary patterns, antibiotic exposure, smoking, infections, medication use, urbanization, and other lifestyle-related factors. Immune dysregulation is characterized by an inappropriate and persistent immune response to intestinal antigens and the gut microbiota. Impaired epithelial barrier function can increase the exposure of immune cells to microbial products, promoting activation of innate and adaptive immune pathways (Figure 1) [3,4].

The gut microbiota is an ecosystem composed of microorganisms, bacteria, archaea, viruses, fungi, and protozoa that colonize the human gut. It plays a role in regulating the immune system and maintaining the integrity of the intestinal barrier. In recent years, there has been an intense surge in research on the composition of the gut microbiota and the metabolites it produces, such as short-chain fatty acids (SCFAs), bile acids (BAs), and tryptophan metabolites [5].

Gut microbiota metabolites comprise a broad group of bioactive molecules, including BA metabolites, tryptophan metabolites, and SCFAs, that are the primary focus in the scientific literature due to their significant effects. Their diversity depends on the composition of the gut microbiota, the availability of dietary substrates, and interactions between microorganisms and the host. Changes in their activity and concentration may potentially reflect functional disturbances in the gut microbiota and be associated with damage to the intestinal barrier, the severity of inflammatory processes, and disease progression [6]. Gut microbiota metabolites can be classified based on their origin, chemical structure, or biological function [6,7]. Among the most extensively studied gut microbiota metabolites to date are BAs, SCFAs, and tryptophan metabolites [7,8,9].

A biomarker is defined as a measurable indicator of biological or pathogenic processes, or of the organism’s response to treatment [10]. Biomarkers are used in the diagnosis of diseases, predicting their course, assessing disease activity, and monitoring response to treatment. In IBD, commonly used biomarkers include C-reactive protein (CRP) levels and erythrocyte sedimentation rate (ESR), measured in blood, and fecal calprotectin, which is a neutrophil-derived S100-family protein with antimicrobial activity and serves as a marker of intestinal inflammation A CRP level of ≥5 mg/L and a fecal calprotectin level of ≥150 µg/g have been reported as thresholds associated with inflammatory activity in IBD [11,12]. Biomarkers of the gut microbiota may serve as promising diagnostic tools, especially for early detection, potentially helping to reduce the risk of disease-related complications. Some studies suggest that changes in the composition of the gut microbiota in patients with IBD may be associated with chronic activation of the immune system [13,14]. The most commonly observed changes related to the gut microbiota include a decrease in SCFA concentrations, particularly butyrate, a reduction in the concentration of tryptophan metabolites that activate the aryl hydrocarbon receptor (AhR), a decrease in the amount of secondary bile acids [8], and an increase in the concentration of pro-inflammatory metabolites [5]. Activation of AhR triggers the expression of specific target genes in host cells, thereby modulating cellular signaling, immune responses, epithelial barrier integrity, and inflammatory processes [8]. The increase in pro-inflammatory metabolites is associated with the accumulation of microbial metabolites that may promote intestinal inflammation and impair intestinal barrier function, thereby contributing to the persistent inflammatory response observed in IBD [5]. This narrative review is based on a literature search conducted in PubMed and Scopus and includes publications available through May 2026. It focuses on short-chain fatty acids, bile acid metabolites, and tryptophan metabolites in inflammatory bowel disease, with particular emphasis on their potential applications in diagnosis, assessment of disease activity, prediction of treatment response, and therapeutic strategies. Evidence from original studies, systematic reviews, and meta-analyses is considered, with study design, sample size, biological material, disease phenotype and activity, treatment context, and study limitations discussed where relevant to the interpretation of individual findings. Throughout the review, preclinical and mechanistic findings are distinguished from human clinical evidence where relevant, and limitations concerning their translation into clinical practice are highlighted.

## 2. Short-Chain Fatty Acids in Inflammatory Bowel Disease

Metabolites produced by gut microbiota are generated primarily during the anaerobic fermentation of dietary fiber, resistant starch, and other undigested carbohydrates that reach the large intestine [15]. In the context of IBD, SCFAs, particularly acetate, propionate, and butyrate, are of particular importance [5,16]. These compounds function as energy substrates, receptor ligands, and epigenetic modulators [16], and their quantitative profiles are associated with the clinical activity of the disease [17]. These mechanisms have been described in preclinical studies, but translating them to the inflammatory environment of the human intestine remains a challenge [18].

### 2.1. Short-Chain Fatty Acids: Production, Mechanisms of Action, and Changes in Inflammatory Bowel Diseases

**Figure 2:**
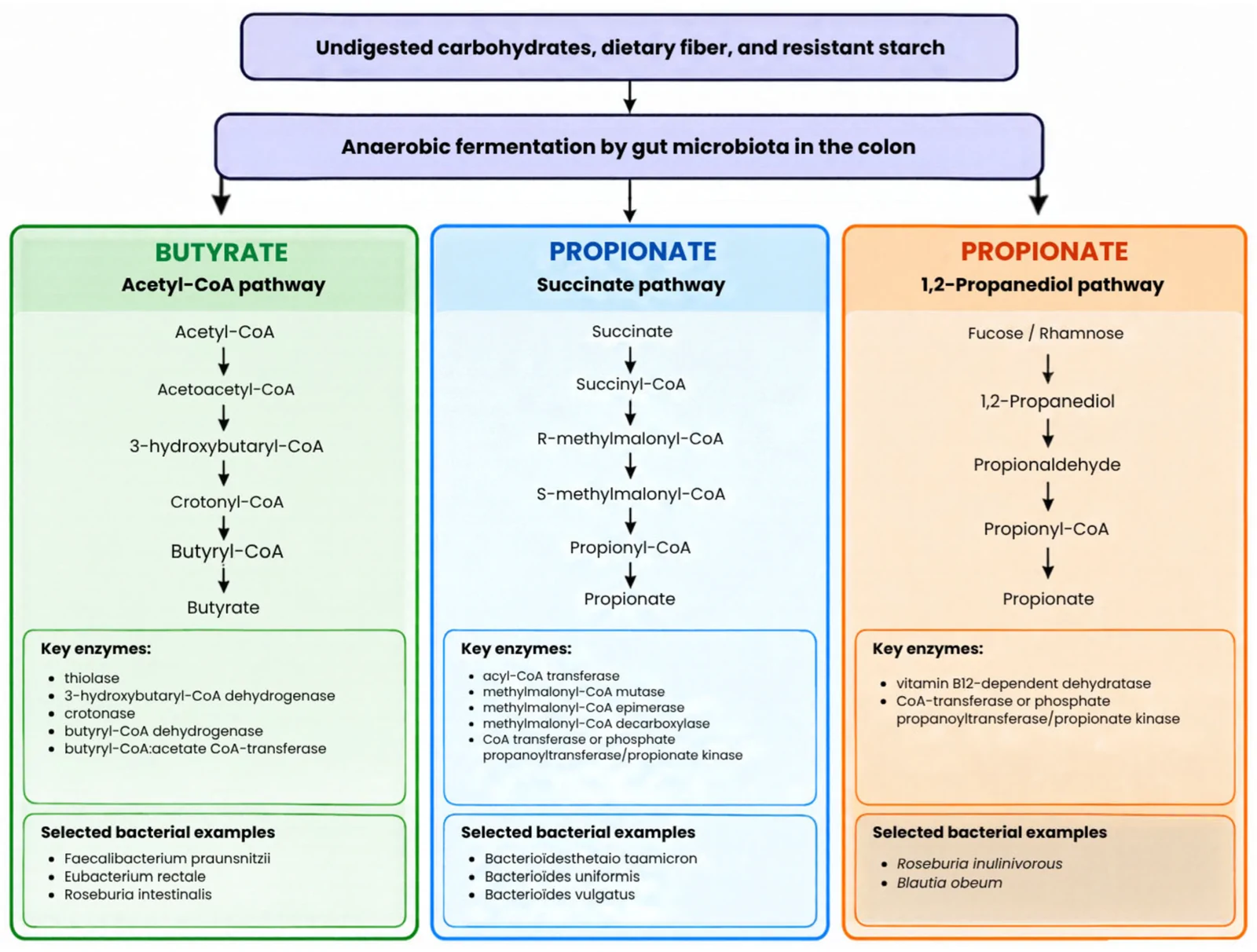
Simplified overview of the microbial pathways of butyrate and propionate production discussed in Section 2.1.

At IBD onset, fecal bacterial alpha diversity is reduced in both CD and UC compared with healthy controls, while pooled bioinformatic reanalysis of treatment-naïve cohorts shows a broad depletion of obligate anaerobes and enrichment of aerobic, facultative anaerobic, microaerophilic, and oral-associated genera. Among the depleted genera, *Alistipes*, *Roseburia*, and *Phascolarctobacterium*, all known SCFA producers, were reduced in both CD and UC compared with both healthy and symptomatic controls [19]. Prominent colonic butyrate producers include *Faecalibacterium prausnitzii* [15,20], *Roseburia* spp., and *Eubacterium rectale* [15], although butyrate production extends across a broader range of bacterial taxa and metabolic pathways [15]. The acetyl-CoA pathway is the predominant butyrate production pathway in the human colonic microbiota, and butyryl-CoA:acetate CoA-transferase is the predominant terminal enzyme among bacteria harboring this pathway [21]. Propionate is produced mainly via the succinate pathway by bacteria of *Bacteroidota*, including *Bacteroides* spp. [15,22], as well as by members of the family Lachnospiraceae in the propanediol pathway [15]. Acetate is the most abundant SCFA in the large intestine, serving as the end product of fermentation by most anaerobic bacteria [15]. The acetyl-CoA pathway of butyrate production and the succinate and 1,2-propanediol pathways of propionate production, together with selected bacterial examples and key enzymes, are summarized in Figure 2.

SCFAs have been shown to exert biological effects through two main mechanisms of action. The first involves the activation of G protein-coupled receptors: free fatty acid receptor 2 (FFAR2) and free fatty acid receptor 3 (FFAR3), which are activated by acetate, propionate, and butyrate, and hydroxycarboxylic acid receptor 2 (HCAR2; GPR109A), which is activated by niacin [23,24] and butyrate [25]. Both FFAR2 and FFAR3 signal through Gi/o proteins, thereby inhibiting adenylate cyclase and lowering cAMP levels, whereas FFAR2 additionally activates Gq proteins, stimulating phospholipase C and increasing intracellular calcium levels [26]. HCAR2 couples to pertussis toxin-sensitive Gi/o proteins [23,24,25]. Available preclinical evidence suggests that acetate can exert opposing, cell-type-specific effects on NLRP3 signaling in colonic epithelial cells it activates the NLRP3 inflammasome via GPR43, whereas in macrophages it attenuates NLRP3 activation and IL-1β production by promoting NLRP3 ubiquitination and autophagic degradation [27,28]. In a DSS-induced mouse model, IL-18 pretreatment reduced inflammation and preserved goblet-cell function via IL-22/STAT3 signaling, whereas administration at later stages exacerbated inflammation and impaired goblet cells [29]. In a complementary genetic model, epithelial-specific deletion of *Il18* or *Il18r1* protected mice from DSS-induced colitis, whereas increased IL-18 bioavailability following *Il18bp* deletion promoted goblet-cell loss and barrier disruption [30]. These findings do not establish the clinical utility of IL-18/IL-18R1 targeting in IBD. A similar translational challenge applies to the HCAR2 pathway.

Preclinical data indicate that HCAR2/GPR109A signaling promotes anti-inflammatory properties in colonic macrophages and dendritic cells, enabling them to induce Treg and IL-10-producing T cells [31]. Supplementary in vitro data in humans indicate that butyrate conditions monocyte-derived dendritic cells (moDCs) to prime naïve CD4^+^ T cells toward IL-10-producing type 1 regulatory T cells (Tr1), through mechanisms requiring both HCAR2 signaling and histone deacetylase (HDAC) inhibition [32]. HDAC inhibition represents an important epigenetic mechanism of SCFA action, increasing histone acetylation and thereby modulating gene expression [18]. This effect is not restricted to a single locus, as butyrate- and propionate-induced hyperacetylation has been observed across multiple histone sites and cell types [33]. Histone hyperacetylation was detectable at SCFA concentrations as low as 10 µM, while 1 mM, used in the main experiments, is within the reported physiological colonic range (0.5–15 mM) but above typical extra-intestinal concentrations [33]. At low concentrations, butyrate and propionate can also enhance histone acetylation independently of substantial HDAC inhibition through their conversion to acyl-CoAs, which promote auto-acylation and activation of p300 [33]. Furusawa et al. [34] identified colonic Tregs as Foxp3^+^ cells within CD3ε^+^CD4^+^ populations by flow cytometry; neuropilin-1− and Helios− Treg populations were considered peripherally generated, whereas CD103 was used as a marker of activated Tregs. Butyrate-induced Treg differentiation was accompanied by increased histone H3 acetylation at the Foxp3 promoter and conserved non-coding sequence 3 (CNS3) before Foxp3 induction, while acetylation at conserved non-coding sequence 1 (CNS1) increased gradually during Treg differentiation [34]. CNS1 and CNS3 are intragenic enhancer elements within the Foxp3 locus; CNS1 is important for peripheral Foxp3 induction, whereas CNS3 enhances the probability of Foxp3 induction [34]. Arpaia et al. [35] further showed that butyrate-induced peripheral Treg generation was CNS1-dependent, as butyrate failed to increase Foxp3^+^ CD4^+^ T-cell populations in CNS1-deficient mice, as assessed by flow cytometry. A similar CNS1-dependent increase in peripheral Tregs was observed following propionate administration, whereas propionate-induced accumulation of colonic Tregs was CNS1-independent [35]. Experimental data [36] and a review [37] indicate that SCFAs, particularly butyrate, promote Treg differentiation while inhibiting the Th17 lineage. In the context of excessive Th17 activation, reduced IL-17 signaling may limit neutrophil recruitment and thereby attenuate intestinal inflammation [37]. Propionate also reduces STAT1 and STAT3 phosphorylation in human CD4^+^ T lymphocytes, thereby decreasing the production of IFN-γ and IL-17 [38].

In human peripheral blood mononuclear cells, butyrate and propionate reduce LPS-induced TNF-α production and NF-κB activation, similarly to trichostatin A, a classic HDAC inhibitor [39]. Data from biopsies and cellular models of patients with IBD indicate that butyrate’s anti-inflammatory effects primarily involve monocytes and macrophages and are associated with HDAC3 inhibition, while its effects in primary human monocytes are independent of GPR109A [40].

Butyrate also plays an important metabolic role in the intestinal epithelium, serving as the preferred energy substrate for differentiated colonocytes in the surface zone [41,42]. In Roediger’s classic 1980 study on isolated human colonocytes ex vivo (colectomy tissue, *n* = 14), butyrate accounted for 73–75% of oxygen consumption as the sole substrate and 59–72% in the presence of glucose [43].

This high oxygen consumption reflects mitochondrial β-oxidation of butyrate, which contributes to physiological hypoxia in the colonic epithelium [44,45,46]. Representative reported pO_2_ values illustrate the intestinal oxygen gradient: pO_2_ is <10 mmHg in the small intestinal lumen, approximately 59 mmHg in the small intestinal wall and 22 mmHg at the villus tip, whereas the sigmoid lumen reaches approximately 3 mmHg [46]. Within the colon, pO_2_ ranges from approximately 5–10 mmHg in tissue adjacent to the lumen to 36–42 mmHg in the vascularized submucosa and 42–71 mmHg in the muscle wall [46]. The distal colonic lumen is therefore near-anoxic. In mouse models, maintenance of low luminal oxygen bioavailability limits the expansion of facultative anaerobic Enterobacteriaceae [45,47]. Epithelial hypoxia stabilizes hypoxia-inducible factor (HIF) by limiting HIF prolyl hydroxylase (PHD) activity. PHDs are Fe^2^^+^- and 2-oxoglutarate (2-OG)-dependent di-oxygenases that use molecular oxygen to hydroxylate proline residues within the oxygen-dependent degradation domain of HIF-α, while converting 2-OG to succinate and CO_2_. Hydroxylated HIF-α is recognized by von Hippel–Lindau protein (pVHL), leading to its polyubiquitination and proteasomal degradation; when PHD activity is reduced, HIF-α is stabilized and can heterodimerize with HIF-1β [48]. PHD2 is the most abundant PHD isoform and a principal regulator of HIF, particularly HIF-1α [48]. Butyrate was shown to bind directly to and noncompetitively inhibit the recombinant PHD2 catalytic domain (PHD2_181−402_), providing an oxygen-consumption-independent mechanism of HIF stabilization [48]. This mechanism was further supported in intestinal epithelial cells and mouse colon tissue in vivo [48]. In patients with IBD, fecal SCFA concentrations show disease- and activity-dependent alterations. A meta-analysis by Chulenbayeva et al. [17] found significantly lower fecal butyrate (overall standardized mean difference (SMD) = −1.37, 95% CI −1.93 to −0.81) and acetate (SMD = −1.07, 95% CI −1.45 to −0.69) in IBD compared with healthy controls. Propionate was significantly lower in UC than in CD (SMD = −0.76, 95% CI −0.95 to −0.57) [17]. Zhuang et al. [49] reported markedly lower fecal butyrate in active UC (SMD = −1.99, 95% CI −3.39 to −0.60) but higher levels during remission (SMD = 0.72, 95% CI 0.34 to 1.11). Xu et al. [50] reported similar estimates for active UC (SMD = −2.09, 95% CI −3.56 to −0.62) and remission (SMD = 0.71, 95% CI 0.33 to 1.10). Chulenbayeva et al. [17] also found that dietary interventions increased fecal butyrate (SMD = 1.03, 95% CI 0.52 to 1.54), whereas effects on acetate and propionate were less consistent; this analysis included only two intervention studies. This disease-activity-dependent pattern was supported by Xu et al. [50], who reported a similarly large reduction in active disease. Notably, both meta-analyses showed an inverse pattern in UC patients in remission, with fecal butyrate concentrations higher than in healthy controls [49,50]. In CD, butyrate-producing taxa, including *Faecalibacterium prausnitzii* and *Roseburia* spp., are less abundant [51,52]. These results should be interpreted with caution, as the concentration of metabolites in feces reflects the dynamic balance of their production, absorption, and cellular consumption [53,54,55]. However, the diagnostic utility of fecal SCFAs remains uncertain. In a single-center study of 64 patients with IBD and 17 healthy controls, fecal acetate, propionate, and butyrate did not differ significantly between IBD and controls, and SCFA levels did not discriminate CD from UC or provide added value for monitoring disease activity [53].

Although the present section focuses on microbiota-derived SCFAs, long-chain fatty acids (LCFAs) may also be relevant modulators of intestinal inflammation in IBD. Oleic acid has been associated with beneficial effects on intestinal inflammation, while *n*-3 polyunsaturated fatty acids, including EPA and DHA, have shown anti-inflammatory and barrier-protective effects predominantly in experimental models. In contrast, *n*-6 polyunsaturated fatty acids are more often associated with pro-inflammatory signaling and impaired barrier function, whereas the effects of saturated LCFAs appear heterogeneous and depend on the specific fatty acid and experimental context [56].

### 2.2. Therapeutic Strategies

Previous attempts at direct SCFA supplementation in patients with IBD have yielded inconclusive results, as summarized by Serrano-Fernández et al. [57] in a systematic review and meta-analysis encompassing ten randomized controlled trials (RCTs) in patients with active UC. SCFA supplementation was not associated with any reported adverse effects, but its clinical efficacy remained inconclusive. Oral SCFAs, evaluated in two studies, reduced calprotectin and CRP levels, while a meta-analysis of rectal infusions showed a non-significant trend toward improvement in clinical activity and histological findings. The authors emphasize that, given the limited number and quality of available studies, SCFAs should currently be considered a potential adjunctive therapy rather than an established intervention. Against this background of inconclusive results, Firoozi et al. [58] (*n* = 36, active UC) demonstrated that 12-week supplementation with sodium butyrate (600 mg/d) significantly reduced fecal calprotectin and high-sensitivity C-reactive protein (hs-CRP) levels compared to placebo. High statistical significance for the reduction in both parameters (*p* < 0.001) was confirmed in the final multiple regression model, adjusted for baseline values as well as the patients’ age and sex. In this study, the formulation was classified as enteric-coated solely based on the manufacturer’s declaration, without measuring butyrate levels in stool to confirm delivery to the colon. The authors themselves acknowledge this as a limitation.

In interpreting these results, it is worth considering that the potential effect of oral butyrate may depend not only on the administered dose, but also on the site and manner of its release in the gastrointestinal tract. Unencapsulated sodium butyrate is largely absorbed already in the proximal small intestine. In a jejunal perfusion study, Schmitt et al. [59] demonstrated that acetate, propionate, and butyrate are absorbed rapidly and with comparable efficiency at this level of the gastrointestinal tract. Roda et al. [60], using a ^13^C-butyrate breath test, showed that uncoated sodium butyrate tablets reached peak ^13^CO_2_ excretion within 30–45 min, whereas the shellac coating delayed this peak by 2–3 h, consistent with release in the ileocecal region. As Caban et al. [61] point out in their review article, uncoated sodium butyrate formulations do not ensure delivery of an adequate amount of the substance to the colon. The lack of measurements of local tissue exposure makes it impossible to assess whether orally administered doses can achieve concentrations effective in inhibiting HDAC3 in vivo. Fecal SCFA concentrations should therefore not be interpreted as a direct measure of local exposure at the colonic mucosal surface. Adequate delivery of SCFAs to their sites of action has been identified as a major limitation of their therapeutic application [18], suggesting that insufficient exposure at the relevant intestinal site may contribute to the inconsistent clinical efficacy of direct SCFA supplementation. This mechanism, which requires direct exposure to butyrate (2 mmol/L), has been demonstrated under ex vivo conditions in cultures of intestinal mucosal biopsies obtained from patients with IBD, as well as in isolated monocytes and macrophages [40]. In contrast, the potential systemic effect is strongly limited by intensive metabolism in the intestinal epithelium and liver. According to the results of an isotopic study conducted in healthy volunteers, the systemic bioavailability of butyrate derived from the colon is only about 2% [62]. In a multicenter, double-blind, randomized, placebo-controlled trial, Karłowicz et al. [63] demonstrated the efficacy of microencapsulated sodium butyrate (MSB; 600 mg/d for 8 weeks) as an add-on therapy in patients with mild-to-moderate active UC. In the intention-to-treat analysis, clinical remission was achieved in 29.6% of patients receiving MSB versus 5.7% receiving placebo (*p* = 0.001), while endoscopic improvement occurred in 22.2% versus 3.77%, respectively (*p* = 0.004). In the per-protocol analysis, corresponding rates were 31.4% versus 6.4% for clinical remission (*p* = 0.004) and 25.5% versus 4.6% for endoscopic improvement (*p* = 0.006). Among patients who achieved clinical remission, changes in fecal butyrate concentration were strongly and positively correlated with changes in the Total Mayo Score (*rho* = 0.80) and endoscopic Mayo Score (*rho* = 0.87). These associations should be considered hypothesis-generating and do not establish causality or provide direct mechanistic evidence. The most important data from clinical trials on SCFA supplementation in IBD are presented in Table 1.

### 2.3. Personalization of Interventions

The therapeutic response to SCFA-based interventions may depend on the patient’s baseline microbiota profile. In a randomized, double-blind, placebo-controlled trial (*n* = 140), Facchin et al. [64] identified two main enterotypes present in both the CD and UC groups. In patients with CD belonging to Enterotype 1 (low *Bacillota*/*Bacteroidota* ratio), supplementation with microencapsulated sodium butyrate formulated using Lipophilic Spray Cooling (LSC) technology was associated with a significantly greater increase in this ratio and a more pronounced improvement in quality of life compared with Enterotype 2 (higher *Bacillota*/*Bacteroidota* ratio), with a non-significant trend toward improvement in clinical activity. The improvement in fecal calprotectin levels did not differ significantly between enterotypes, which the authors attributed to the small size of the subgroups. These results indicate that Enterotype 1 may represent a population more susceptible to modulation of the gut microbiota by microencapsulated sodium butyrate; however, this observation is based on a small subgroup of patients with CD and Enterotype 1 (*n* = 26; microencapsulated sodium butyrate, *n* = 14; placebo, *n* = 12) and requires confirmation in a prospective study with objective endpoints before the concept of stratification can be applied clinically. A limitation of this approach is the sensitivity of the *Bacillota*/*Bacteroidota* ratio to the choice of pipeline, which modifies the estimated relative abundance [65]. In multicenter settings, interlaboratory variance exceeds interindividual variance [66]. It has been independently confirmed that the choice of pipeline takes precedence over the library preparation protocol and the amplicon region [67], which necessitates standardization of the methodology prior to the clinical implementation of stratification [68].

Beyond reliable microbiome classification, however, it remains crucial to determine whether dietary interventions can effectively increase SCFA concentrations in patients with IBD. In a meta-analysis by Chulenbayeva et al. [17], based on two intervention studies in patients with UC, the dietary interventions included increasing the intake of fermentable fiber while restricting protein in one study and consuming a fermented vegetable beverage in the other. These interventions were associated with a statistically significant increase in butyrate levels, whereas increases in acetate and propionate levels did not reach statistical significance. In patients whose gut microbiota has an impaired ability to ferment fiber, unfermented β-fructans may exacerbate the inflammatory response through NLRP3- and TLR2-dependent pathways. Armstrong et al. [69], using a model of ex vivo-cultured colon biopsies from children with IBD, demonstrated that intact β-fructans increased the secretion of pro-inflammatory cytokines in a subgroup of patients with active disease, whereas fermentation by the gut microbiota from healthy individuals or patients in remission abolished this effect. These results do not call into question the overall role of dietary fiber in the nutrition of patients with IBD, but they suggest that its potential effects may depend on the clinical context and the gut microbiota’s ability to ferment it. The 2024 American Gastroenterological Association (AGA) Clinical Practice Update advises a Mediterranean diet for overall health in IBD unless contraindicated, considers a low-residue or low-fiber diet reasonable during symptomatic flares, and states that long-term management should attempt to reintroduce fiber, preferably soluble fiber, with a return to a Mediterranean-style diet after symptom resolution [70].

However, the metabolic efficacy of such reintroduction is not guaranteed, as the butyrate response to fiber supplementation varies from person to person, and the baseline composition and functional potential of the gut microbiota remain key determinants of this response [71]. In a 2-week intervention study by Baxter et al. [72] involving 174 healthy young adults, resistant potato starch increased fecal butyrate concentrations by an average of 29%, although responses varied markedly between individuals. Higher fecal butyrate during supplementation was observed in 76% of participants with detectable *Ruminococcus bromii* at baseline, compared with 36% of those without detectable *R*. *bromii*. Changes in *R. bromii* abundance were positively correlated with changes in the abundance of the butyrate-producing species *Eubacterium rectale*, whose relative abundance was also positively correlated with fecal butyrate concentration during resistant potato starch supplementation. Inulin supplementation also increased the abundance of the butyrate producers *Anaerostipes hadrus* and *E. rectale* but did not significantly increase fecal butyrate, indicating that changes in the abundance of butyrate-producing bacteria and fecal butyrate concentrations are not necessarily concordant [72]. In humanized mouse models, multigenerational exposure to a low-fiber diet induces a loss of gut microbial diversity that is not fully reversed by reintroduction of dietary fiber alone [73]. In the context of IBD, patients with advanced depletion of butyrate-producing bacteria may therefore not restore butyrate production simply by increasing dietary fiber intake without simultaneous recolonization with the appropriate species, which justifies the search for strategies aimed at directly restoring the missing bacterial species. *F. prausnitzii* exhibits high sensitivity to oxygen, as colonies of the parent strain DSM 32186 were not recovered after 20 min of exposure to atmospheric air [74]. This supports the need for protection against oxygen and premature release in the acidic environment of the stomach following oral administration. Guarino-Vignon et al. [75] published the results of the first-in-human clinical trial involving oral administration of the *F. prausnitzii* EXL01 strain in adults with mild to moderate CD with ileal involvement who achieved corticosteroid-induced clinical response or remission, in the open-label, single-arm Part A of a Phase I trial (the planned randomized Part B was discontinued due to low recruitment; n = 8). No EXL01-related treatment-emergent adverse events were reported, although two participants discontinued treatment due to disease flare. Transcriptomic analyses of ileal biopsies revealed modulation of immune-related genes and upregulation of energy metabolism pathways following EXL01 treatment. The two participants who experienced disease flare were characterized by higher baseline expression of innate immune genes and markers of systemic inflammation. Concurrently, Danne et al. [76] demonstrated in an ex vivo/in vitro study that *F. prausnitzii* EXL01 induces a dose-dependent production of IL-10, with CD14^+^ monocytes being the main producers, in cells from the peripheral blood of healthy volunteers and the intestinal lamina propria of patients with IBD and noninflamed controls, without inducing the pro-inflammatory response observed with LPS. Furthermore, RNA-seq and Seahorse analyses revealed that *F. prausnitzii* EXL01 reprograms the energy metabolism of monocytes in a manner distinct from that of *Coprococcus comes*, *Escherichia coli*, and LPS, and that the anti-inflammatory response was dependent on mitochondrial respiration. These results provide the first mechanistic insights into the anti-inflammatory effects of *F. prausnitzii* at the cellular level, although their clinical relevance and translation require further validation.

In conclusion, the efficacy of SCFA-based interventions in IBD is strongly linked to both the form in which the metabolite is delivered and the baseline composition of the patient’s microbiota. The butyrate response to fiber supplementation is individualized and depends on the microbiota’s ability to increase the abundance of key primary degraders and their associated butyrate producers, a phenomenon demonstrated so far only in a population of healthy volunteers [72]. Microbiome-based stratification according to the *Bacillota*/*Bacteroidota* ratio may offer an approach to identify patients with CD according to their potential response to butyrate supplementation [64], whereas the development of *F. prausnitzii*-based live biotherapeutics represents a distinct microbiome-targeted therapeutic strategy, supported by preliminary safety and tolerability data [75] and mechanistic evidence of an anti-inflammatory effect in human CD14^+^ monocytes [76]. Together, these approaches may provide complementary avenues for personalized microbiome-informed interventions in IBD, although their combination has not yet been evaluated.

## 3. Bile Acids

### 3.1. Microbial Transformation of Bile Acids (Primary vs. Secondary)

Bile acids are a group of steroid compounds produced in the liver through the metabolism of cholesterol, including its oxidation catalyzed by enzymes of the cytochrome P450 (CYP) superfamily [77,78]. The primary physiological role of bile acids is to emulsify fats and facilitate the absorption of fats and fat-soluble vitamins. In addition, they act as signaling molecules involved in regulating metabolism, maintaining proper intestinal barrier function, and modulating immune system activity through receptors such as FXR, VDR and TGR5. The secondary bile acids lithocholic acid (LCA) and deoxycholic acid (DCA) activate TGR5, thereby triggering the cAMP/PKA signaling pathway, which may inhibit NLRP3 inflammasome activity and consequently attenuate inflammatory responses. TGR5 signaling may also promote intestinal epithelial regeneration via the SRC/YAP pathway. FXR regulates genes involved in bile acid homeostasis and the maintenance of intestinal barrier integrity, whereas LCA-mediated activation of VDR may suppress Th1-dependent inflammatory responses [78,79]. The primary bile acids found in humans are cholic acid (CA) and chenodeoxycholic acid (CDCA). They are synthesized in hepatocytes and then conjugated primarily with glycine or taurine, which improves their hydrophilic properties and enables effective secretion into bile [80,81]. Once released into the lumen of the small intestine, bile acids play a role in the digestion and absorption of lipids. Most of them are subsequently reabsorbed in the terminal ileum and transported back to the liver via the enterohepatic circulation. The remaining portion, which has not been absorbed, enters the large intestine, where it undergoes further metabolic transformations [78,79,81].

Primary bile acids are produced directly by the host organism, whereas secondary bile acids are formed by the action of enzymes produced by microorganisms colonizing the intestine. Through its metabolic activity, the gut microbiota significantly increases the chemical diversity of bile acids [82]. Bacteria colonizing the gut catalyze a series of transformations. These include deconjugation [83], 7α-dehydroxylation [84], oxidation, and epimerization [82,85]. The key reaction is 7α-dehydroxylation, in which CA is converted to deoxycholic acid (DCA), and CDCA to lithocholic acid (LCA). This process depends on the *bai* operon and is carried out by a limited group of bacteria, including *Clostridium scindens*, *Clostridium hylemonae*, and *Peptacetobacter hiranonis* [84]. DCA and LCA can subsequently undergo oxidation and epimerization catalyzed by hydroxysteroid dehydrogenases, leading to the formation of derivatives such as 3-oxo-DCA, 3-oxo-LCA, isoDCA, allo-LCA, and isoallo-LCA [85,86,87].

These metabolites exhibit varying degrees of biological activity. In a study, Campbell et al. [88] demonstrated that isoDCA promotes the formation of Treg cells by limiting the immunostimulatory properties of dendritic cells. The immunological effect of the bile acid depends not only on the levels of DCA and LCA but also on the presence of their more specialized derivatives. In patients with IBD, these metabolic processes are impaired. In UC, lower concentrations of DCA and LCA, higher concentrations of CDCA, and reduced expression of *bai* operon genes are observed. The bile acid-inducible (*bai*) operon encodes enzymes required for the 7α-dehydroxylation of primary bile acids and their conversion into secondary bile acids, including DCA and LCA. This metabolic capacity is relatively rare and is mainly found among members of the Ruminococcaceae and Lachnospiraceae families within *Clostridium* cluster XIVa [79]. There is also a strong association between dysbiosis and changes in the fecal metabolome [89] as well as reduced levels of 3-oxo-DCA, 3-oxo-LCA, allo-LCA, isoallo-LCA, and 3-oxo-UDCA [90]. IBD is associated not only with changes in the composition of the gut microbiota but also with a loss of its metabolic functions, leading to an increase in the proportion of primary bile acids and a decrease in the proportion of secondary bile acids [79,80,90].

### 3.2. Signaling via the Farnesoid X Receptor (FXR) and the G Protein-Coupled Bile Acid Receptor 1 (TGR5) and Their Effects on Inflammation and the Intestinal Barrier

Bile acids act as signaling molecules, helping to maintain intestinal homeostasis. This regulation occurs through the activation of the nuclear receptor FXR and the membrane receptor TGR5. Upon ligand binding, FXR regulates the transcription of genes involved in bile acid homeostasis, epithelial barrier integrity, and inflammatory responses, whereas TGR5, as a G protein-coupled receptor, activates intracellular cAMP/PKA-dependent signaling pathways. FXR activation can stabilize the epithelial barrier, reduce the production of pro-inflammatory cytokines, and regulate the activity of immune cells [91,92,93]. TGR5, on the other hand, participates in regulating epithelial activity, inhibiting enterocyte apoptosis, and limiting the inflammatory response, partly through signaling pathways involved in epithelial regeneration and cell survival [94,95,96]. Chenodeoxycholic and lithocholic acids activate FXR and TGR5, respectively, which in turn may affect the integrity of the intestinal barrier, limit the inflammatory process, and modulate the response to biologic therapy [97].

Available observational studies in humans examining FXR and TGR5 signaling in IBD focus primarily on bile acid profiles [98,99,100], receptor expression [101] and fibroblast growth factor 19 (FGF19) levels as a surrogate marker of FXR activation [98,102,103]. Under physiological conditions, activation of ileal FXR by bile acids induces FGF19 secretion, which acts on the liver to suppress CYP7A1-mediated bile acid synthesis through negative feedback. In IBD, this axis appears to be impaired: patients with CD show reduced FXR activation and significantly lower circulating FGF19 levels, while in UC lower FGF19 concentrations are associated with higher inflammatory marker levels and greater disease activity. The results indicate a disruption of the bile acids–FXR/TGR5 axis in CD and UC and its association with the severity of inflammation; however, the causal significance of these changes and their impact on the intestinal barrier have not been confirmed in human interventional studies [98,99,100,101,102,103]. Wilson et al. [98] demonstrated a variable profile of plasma bile acids in 74 patients with CD, including, among other things, a lower proportion of glycocholenic, taurocholic, and lithocholic acids. These profiles activated FXR to a lesser extent than those of healthy individuals, and FGF19 concentrations were significantly lower, indicating an impairment of the FXR–FGF19 axis in CD. The association between FGF19 and inflammatory activity has also been described in patients with UC. FGF19 levels tended to be lower in active UC than in inactive UC; however, the difference was not statistically significant. However, inverse correlations between FGF19 and fecal calprotectin and CRP levels, as well as the severity of abdominal pain, were statistically significant [102]. In contrast, a study of a larger group of patients with IBD showed that higher FGF19 levels were observed during remission than during active phases of UC and CD. The concentration of this factor was inversely correlated with clinical and endoscopic disease activity, as well as with the severity of diarrhea, abdominal pain, and selected inflammatory markers [103]. The results of both studies support an association between the FXR–FGF19 axis and IBD activity, but do not prove that reduced FGF19 is the primary cause of inflammation [102,103].

Studies in animal models have shown that FXR plays a significant role in maintaining the integrity of the intestinal barrier and regulating the inflammatory response [91,92]. FXR activation strengthens the tight junctions of the intestinal epithelium, limits damage to the intestinal barrier and reduces the severity of inflammation. These effects were observed, among other things, through increased expression of tight junction proteins (TJPs) such as claudin-1, occludin, and ZO-1, as well as through the inhibition of pro-inflammatory pathways, including the NLRP3 inflammasome [104,105]. Furthermore, FXR influences the activity of intestinal immune cells, including by limiting the pathologically excessive activation of group 3 innate lymphoid cells (ILC3) and the production of pro-inflammatory cytokines. ILC3s are characterized by the expression of RORγt, CD127, and c-Kit, while activated intestinal ILC3s may also express NKp44; functionally, they are associated with the production of IL-17A and IL-22. Research findings indicate that proper FXR activation is an important protective mechanism in diseases characterized by intestinal barrier dysfunction and chronic inflammation [105,106].

Data on TGR5 come, among other sources, from a study by Yang et al. [101], which included 32 patients with active UC and 23 healthy individuals. Patients were found to have elevated levels of selected primary and conjugated bile acids, as well as reduced levels of DCA and LCA, which are TGR5 ligands. The expression of this receptor in the colonic mucosal epithelium was higher than in healthy individuals. Sun et al. [99] demonstrated lower concentrations of secondary bile acids in 62 patients with CD compared to 33 healthy individuals. Concentrations of DCA and glycodeoxycholic acid (GDCA), as well as the ratio of DCA to the sum of DCA and CA, were lower during active disease than during remission and inversely correlated with the Crohn’s disease activity index (CDAI) score. A similar disturbance in the ratio of primary to secondary bile acids has been described in active UC [107]. A study by Xu et al. [100] demonstrated reduced levels of CDCA, DCA, LCA, and their conjugated derivatives. At the same time, the ratio of primary to secondary bile acids was increased, and the DCA concentration alone distinguished patients with IBD from healthy individuals. Ay et al. [108] further confirmed that microbiologically conjugated bile salts present in human bile can activate FXR and TGR5. This may suggest that their effects are more significant locally, in the intestinal lumen or within the enterohepatic circulation, than in the systemic circulation. However, the study did not directly involve patients with IBD; therefore, it provides evidence of the biological activity of human metabolites rather than confirmation of their role in the course of CD or UC.

### 3.3. Changes in Bile Acid Profiles in CD and UC. Clinical Significance

Bile acid profiles are assessed in stool, serum, or plasma samples using targeted and untargeted metabolomics, most commonly via LC–MS/MS. These analyses are increasingly supplemented with metagenomic data and patients’ clinical parameters. In a prospective cohort study, Schirmer et al. [109] demonstrated that the integration of fecal and plasma metabolomics with metagenomics revealed distinct signatures depending on the type of sample and UC activity. Gentry et al. [110] applied reverse metabolomics and validation across four IBD cohorts, demonstrating the utility of new bile acid conjugates in disease phenotyping. Feces more accurately reflect local microbial changes, whereas blood reflects disturbances in the enterohepatic circulation. Therefore, results from these two matrices should not be treated as interchangeable [109,110]. Despite methodological differences, active UC is often associated with an increased proportion of primary and tauroconjugated acids and a decrease in secondary and more specialized derivatives [109,111,112,113]. In severe pediatric UC, an increase in tauroconjugated bile acids and a decrease in amino acid-conjugated bile acids were observed in stool [109]. A reduction in the total pool of FA-isoBA esters, an increase in primary bile acids, and a decrease in secondary bile acids were also demonstrated in children with UC [112]. Following fecal microbiota transplantation (FMT) in children with UC, the proportion of primary bile acids decreased, the secondary-to-primary bile acid ratio approached that observed in healthy controls, and the abundance of genes encoding bile salt hydrolase (BSH), hydroxysteroid dehydrogenases (HSDHs), and enzymes of the *bai* operon increased. BSH catalyzes the deconjugation of bile acids, whereas HSDHs mediate their oxidation and epimerization, thereby contributing to microbial bile acid transformation [113]. The CD profile is more heterogeneous. Gentry et al. [110] demonstrated, across four cohorts, an increase in new CA conjugates with Glu, Ile/Leu, Phe, Thr, Trp, and Tyr. In an analysis of 302 stool samples, Tan et al. [114] found higher levels of cholanate in CD affecting the ileum and the ileocolonic forms compared to disease confined to the colon, as well as higher levels of secondary bile acids in the stenotic and penetrating phenotypes compared to the inflammatory phenotype. In patients initiating anti-TNFα therapy, fecal and serum bile acid profiles predicted treatment response, achieving ROC area under the curve values of 0.81 and 0.74, respectively, while higher concentrations of phase II conjugates were associated with a lack of response. Receiver operating characteristic (ROC) curve analysis evaluates the ability of a biomarker or predictive model to distinguish between responders and non-responders by assessing its sensitivity and specificity across different cut-off values [115]. In patients treated with anti-α4β7-integrin therapy, which blocks α4β7-mediated leukocyte homing to the intestinal mucosa, sustained remission was associated with persistent microbiota-dependent changes in bile acid metabolism, particularly increased levels of secondary bile acids such as lithocholic acid (LCA) and deoxycholic acid (DCA), together with enrichment of *Lactobacillus* and *Clostridium* species [116]. Improvement in the bile acid profile was also observed in 12 pediatric patients with active CD receiving exclusive enteral nutrition (EEN) for 8 weeks, administered orally in 10 patients and via a nasogastric tube in 2 patients [117], as well as in UC following fecal microbiota transplantation (FMT), using either autologous stool or donor stool administered via colonoscopy [113]. Multimetabolite panels, including bile acids such as CA, CDCA, DCA, and LCA, together with acylcarnitines, ceramides, sphingomyelins, methylysine, and polyamines, integrated with microbiota and inflammatory markers, appear to be more promising than single-metabolite approaches. The direction of changes depends on the type of biological material, disease activity and location, history of bowel resections, and the treatment administered. The most consistent abnormality is a decrease in the proportion of DCA, LCA, and their derivatives, accompanied by a relative increase in primary and conjugated bile acids, particularly in active UC (Table 2) [114,118].

Changes in the BA profile reflect inflammatory activity and gut microbiota disturbances. Active inflammation is associated with reduced microbial diversity, depletion of secondary bile acids, and increased tauro-conjugated bile acids, while BA patterns may also vary with disease phenotype and location [109,110,114,118]. In UC, the profile is characterized by limited conversion of primary BA into secondary metabolites, and partial normalization of this profile has been observed following fecal microbiota transplantation [109,112,113]. In CD, greater heterogeneity of lesions may reflect ileal involvement, disease phenotype, and impaired enterohepatic circulation [109,114]. Bile acid profiles may aid in assessing disease activity, phenotyping patients, and predicting and monitoring responses to biologic or dietary therapy [115,116,117]. However, multimetabolite panels combined with microbiological data and markers of inflammation show the greatest potential, rather than individual metabolites. Before these markers can be applied in clinical practice, standardization of assay methods and validation in large, independent cohorts are necessary [109,110,115].

## 4. Tryptophan Metabolites

Tryptophan (TRP) belongs to the group of exogenous amino acids, meaning that it cannot be synthesized by the human body and therefore must be supplied through the diet. It is incorporated into proteins during synthesis in the body, but it also serves as a precursor to metabolites involved in the regulation of neurotransmission, immune and inflammatory response, circadian rhythms and gut homeostasis [119,120,121]. This amino acid is found, among other sources, in chocolate and legume seeds [122], as well as in animal-derived products such as turkey, chicken, beef, pork, fish, eggs, and milk [123]. Many factors influence the concentration of tryptophan in the blood. Among others, cultural factors, diet, and geographic factors play a significant role, as do the levels of physical activity and stress in daily life. These factors may contribute to both an increase and a decrease in blood tryptophan levels [124]. Tryptophan metabolism serves as a crucial link between the gut microbiota and immune system function. Tryptophan metabolites produced by both the human body and gut bacteria modulate immune cell activity through pathways such as aryl hydrocarbon receptor (AhR) signaling, thereby promoting IL-22 production, strengthening the intestinal barrier and supporting gut homeostasis [125].

### 4.1. Tryptophan Metabolism Pathways: Indole Derivatives, Kynurenines, and Serotonin

In addition to the interaction of genetic, immunological, and environmental factors, studies increasingly indicate that tryptophan metabolism disorders also indirectly play a role in the pathogenesis of IBD [126,127,128]. In the human organism, tryptophan, as an exogenous amino acid, can be metabolized via three main pathways. Under physiological conditions, the majority (approximately 95%) is metabolized via the kynurenine pathway (KP) [129]. In the first phase, tryptophan is converted to N-formylkynurenine via tryptophan 2,3-dioxygenase (TDO2), which acts primarily in the liver, or via indoleamine 2,3-dioxygenase 1/2 (IDO1/IDO2), which is present in the brain and immune system cells [129,130,131]. Differentiating between IDO and TDO2 activity may have clinical significance in IBD, as both enzymes initiate the kynurenine pathway but reflect distinct mechanisms of tryptophan metabolism. TDO regulates the kynurenine pathway primarily under physiological conditions, whereas IDO plays a greater role mainly during the immune response [130,132,133]. Intestinal biopsy studies show increased IDO expression in patients with active UC and CD, and decreased TDO2 expression in the study group [132,134]. In the course of IBD, heightened production of pro-inflammatory cytokines may increase IDO expression in the central nervous system, thereby shifting tryptophan metabolism toward the kynurenine pathway [129]. As a result of subsequent transformations, N-formylkynurenine is converted by formamidase into kynurenine, which is the central metabolite of the entire pathway. Through further reactions, kynurenine can be converted into anthranilic acid (AA), kynurenic acid (KA), or 3-hydroxykynurenine (3-HK). 3-HK is further metabolized to 3-hydroxyanthranilic acid (3-HAA) and then to quinolic acid (QA). Ultimately, quinolic acid is used for the synthesis of NAD+ (nicotinamide adenine dinucleotide), which is responsible for cellular energy production [122,135].

A small part of tryptophan (about 1–2%) is converted via the serotonin pathway, which produces serotonin (5-HT), which is responsible, among other things, for regulating mood, appetite, and sleep patterns. These reactions involve, among others, tryptophan hydroxylase (TPH1) and aromatic amino acid decarboxylase (AADC) [125]. The gastrointestinal tract is the primary site of tryptophan metabolism, which includes serotonin synthesis and the production by the gut microbiota of indole derivatives that influence the immune system [122]. In intestinal cells, tryptophan is metabolized by TPH1 into 5-hydroxytryptophan (5-HTP) and then into serotonin. In patients with IBD, there may be reduced expression of TPH1 [136] and inhibition of serotonin synthesis [127]. Through subsequent metabolic reactions, 5-HT is converted to N-acetyl-5-hydroxytryptamine by the enzyme N-acetyltransferase (AANAT). Ultimately, through the action of hydroxyindole-O-methyltransferase (HIOMT), melatonin is formed as the end product of the pathway [135,137], which functions as a neuromodulator with strong antioxidant properties and plays an important role in regulating circadian rhythms and sleep, while also exerting immunomodulatory effects [123].

**Figure 3:**
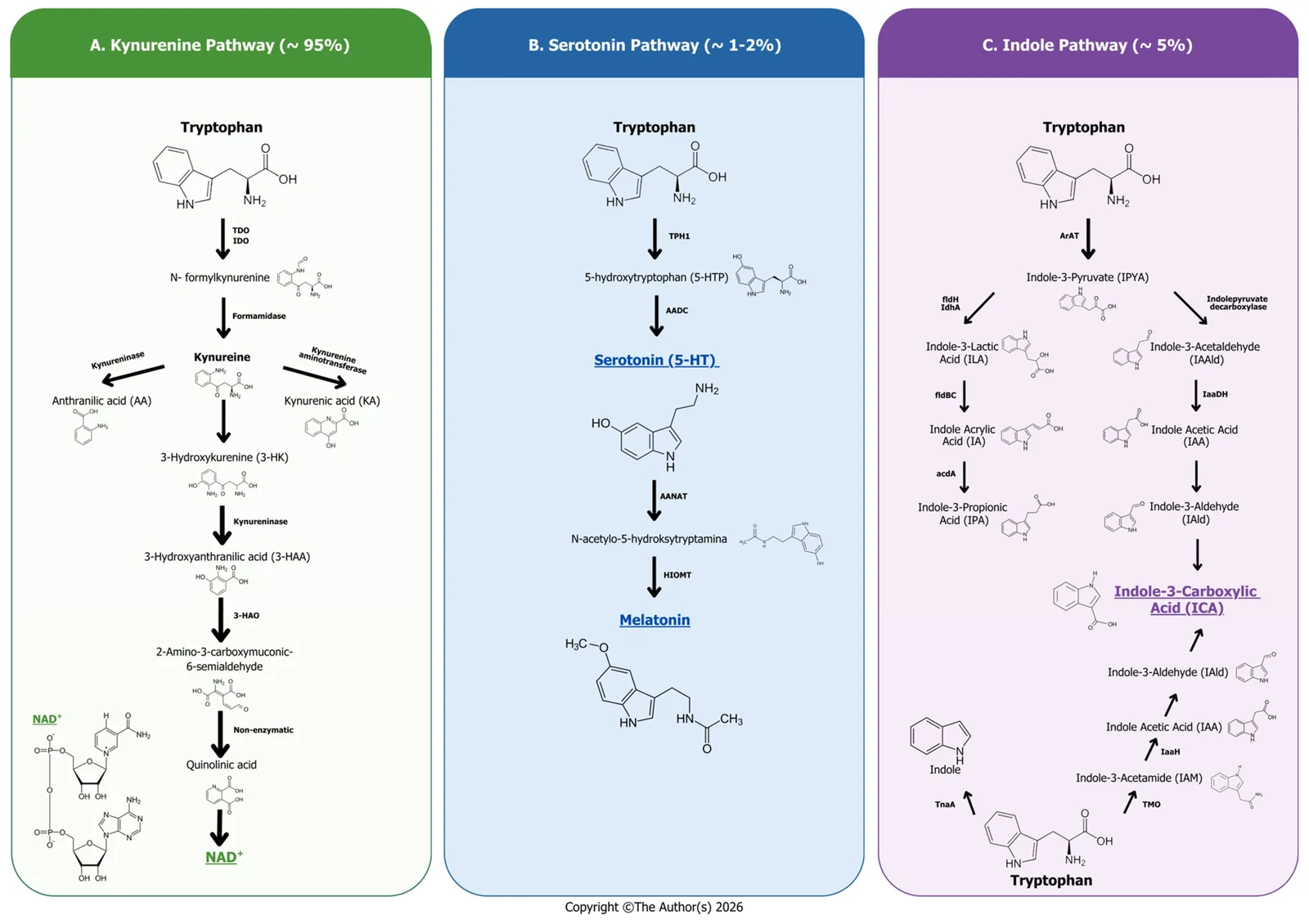
Tryptophan metabolic pathways. Abbreviations: TDO—tryptophan-2,3-dioxygenase; IDO—indoleamine-2,3-dioxygenase; 3-HAO—3-hydroxyanthranilic acid oxygenase; TPH1—Tryptophan hydroxylase; AADC—Aromatic amino acid decarboxylase; AANAT—Arylalkylamine N-acetyltransferase; HIOMT—Hydroxyindole-O-methyltransferase; ArAT—Aromatic amino acid aminotransferase; fldH—Phenyllactate dehydrogenase; IdhA—D-2-hydroxyacid dehydrogenase; fldBC—phenyllactate dehydratase; acdA—acyl-CoA dehydrogenase; IaaDH—Indoleacetaldehyde dehydrogenase; IaaH—Indoleacetamide hydrolase; TMO—Tryptophan 2-monooxygenase.

The gut microbiota, through bacterial enzymes, plays a significant role in tryptophan metabolism, leading to the formation of bioactive indole metabolites. They influence physiological processes by modulating immune responses, maintaining intestinal barrier integrity and regulating inflammation [120,138]. Consequently, alterations in the composition and metabolic activity of the gut microbiota may disturb tryptophan metabolism and intestinal homeostasis, thereby contributing to the development and progression of IBD [139]. Approximately 5% of tryptophan is metabolized into indole and its derivatives, which support intestinal barrier integrity by regulating epithelial activity and modulate the immune system by influencing immune cell activity and inflammatory response [135,139]. Microorganisms involved in tryptophan metabolism include, among others, the bacteria *Bifidobacterium*, *Lactobacillus*, *Clostridium*, *Bacteroides*, *Parabacteroides*, and *Peptostreptococcus* [138]. At the beginning of the pathway, tryptophan is converted to indole-3-pyruvate (IPYA) via aromatic amino acid aminotransferase (ArAT). IPYA can then be metabolized to indole-3-lactic acid (ILA) by phenyl-lactate dehydrogenase (fldH) or D-2-hydroxyacid dehydrogenase (IdhA). In a subsequent step, ILA is converted to indoleacrylic acid (IA) and indole-3-propionic acid (IPA) with the involvement of specific enzymes (fldBC-phenyllactate dehydratase and acdA-acyl-CoA dehydrogenase). Indole-3-pyruvate, in the presence of other enzymes (ID-indolepyruvate decarboxylase), can also be converted to indole-3-acetaldehyde (IAAld) and subsequently to indoleacetic acid (IAA). Through subsequent transformations, IAA can be metabolized into indole-3-aldehyde (IAld) and indole-3-carboxylic acid (ICA). In another branch of the pathway, tryptophan can also be metabolized into indole-3-acetamide (IAM) and subsequently into indoleacetic acid. In the TnaA (Tryptophanase)-dependent branch of the pathway, tryptophan is converted to indole. TnaA is found, among others, in *Escherichia coli* and certain lactic acid bacteria. Indole produced in biochemical reactions can undergo further transformations by the host organism and be converted into derivative compounds (Figure 3) [9,138].

Indole derivatives produced by the gut microbiota act as ligands for the AhR receptor, activating signaling pathways involved in the regulation of mucosal immune responses, maintenance of intestinal barrier integrity, and control of inflammation [140]. In patients with IBD, the production of these ligands by the gut microbiota may be reduced, which can reduce anti-inflammatory mechanisms and contribute to the persistence of inflammation in the intestines. Additionally, patients with UC have been found to have reduced levels of IPA, one of the indole derivatives [139,141].

### 4.2. Role in Immune Modulation and Intestinal Barrier Integrity

The gut microbiota is closely linked to tryptophan metabolism, influencing the progression of IBD [139]. Research findings indicate that patients with IBD have reduced serum tryptophan levels. This applies to patients with both CD and UC [132,134,142,143]. In a study by Nikolaus et al. [132] involving 535 patients with IBD, serum tryptophan concentrations were measured using HPLC (high-performance liquid chromatography). The final analysis showed that patients with IBD had significantly lower tryptophan levels compared to those in the control group. Similar results were reported in a smaller study by Paydaş Hataysal et al. [143] involving 80 patients in IBD remission. Tryptophan levels were significantly lower in both patients with CD and those with UC (CD: 19,700 ng/mL, UC: 19,900 ng/mL, controls: 25,100 ng/mL), which may reflect enhanced tryptophan catabolism through the kynurenine pathway, as indicated by increased IDO activity and an elevated KYN/TRP ratio [132]. In addition, clinical studies show that the lower the serum tryptophan level, the higher the IBD activity and the higher the concentration of C-reactive protein [132,134,142]. Patients with IBD also have lower levels of gut microbiota-derived aryl hydrocarbon receptor (AhR) ligands, particularly IPA and IAA, which may contribute to impaired intestinal barrier integrity and the development of intestinal inflammation [139]. Some metabolites of tryptophan, including indole and its derivatives, can bind to the AhR receptor in the intestines, activating its function [140]. The AhR receptor, present in intestinal epithelial cells, among others, belongs to the PER-ARNT-SIM family of transcription factors and regulates processes involved in intestinal barrier integrity, mucosal immune responses, epithelial cell proliferation and differentiation and inflammatory signaling [140,141]. After activation, AhR migrates to the cell nucleus, where it forms a heterodimer with the ARNT receptor, which facilitates binding of the complex to specific DNA response elements and subsequent regulation of target gene expression [140]. This complex influences the activity of genes involved, among other things, in regulating IL-22 production, which plays a role in modulating the immune response and strengthening the intestinal barrier. IL-22 promotes the expression of antimicrobial peptides and enhances mucin production by goblet cells, thereby contributing to intestinal mucosal defense [140,144,145]. Tryptophan metabolites, through the AhR receptor, can also support the development of cells with anti-inflammatory effects, such as regulatory T cells. This effect is associated with the induction and stabilization of FOXP3, a key transcription factor required for regulatory T cells differentiation and suppressive function. Metabolites can also limit the excessive activity of pro-inflammatory Th17 cells, thereby exerting an anti-inflammatory effect and reducing excessive intestinal inflammation [141,146]. Indole derivatives, such as indole-3-propionic acid and indoleacetic acid, promote intestinal epithelial cell proliferation, limit excessive apoptosis, and thereby help maintain the integrity of the intestinal mucosal barrier [139]. Similarly, host-produced kynurenine may contribute to the maintenance of intestinal barrier integrity through AhR-dependent regulation of IL-10 receptor expression in intestinal epithelial cells. Activation of AhR by kynurenine may increase IL-10 receptor expression, thereby enhancing epithelial responsiveness to IL-10. This, in turn, may promote epithelial repair, reduce inflammation, and support preservation of barrier function [147,148]. Additionally, certain metabolites of the kynurenine pathway, by activating the AhR receptor, can regulate the functions of immune cells [139]. Tryptophan metabolites, including, among others, indole-3-aldehyde, indole-3-pyruvate, and indole-3-ethanol, which are formed in the indole pathway, may directly affect the structure of junctions between intestinal epithelial cells. They support the integrity and organization of tight junctions (TJ) and adherens junctions (AJ), which together form the apical junction complex (AJC), by preserving junctional structure and modulating proteins associated with the actin cytoskeleton. This complex is primarily responsible for regulating intestinal permeability. Indole derivatives stabilize the AJC and proteins involved in the regulation and organization of the actin cytoskeleton, including myosin IIA and ezrin. Reducing their excessive actin cytoskeleton contractility contributes to stabilizing the junctions between epithelial cells, thereby limiting intestinal permeability [147]. Serotonin, which is also one of tryptophan’s metabolites, is primarily responsible for regulating intestinal motility by influencing peristalsis and secretory processes. It also participates in signaling within the enteric nervous system, thereby contributing to the regulation of gastrointestinal function [139].

### 4.3. Clinical Application: Biomarkers and Therapeutic Strategies

Clinical and preclinical studies confirm the link between the microbiota–tryptophan axis, which regulates immune system function, metabolism, and neurotransmission, and is also involved in the pathogenesis of many diseases [120]. Tryptophan metabolites may serve as an adjunctive tool in the diagnosis of IBD. Patients with CD and UC have significantly lower serum tryptophan concentrations, which negatively correlate with disease activity and CRP levels [132]. Differences are also evident in the levels of tryptophan metabolites between healthy individuals and those with newly diagnosed IBD, particularly in the case of kynurenine metabolites [149]. Quinolinic acid, which is produced in the kynurenine pathway, may also serve as a valuable biomarker to aid in diagnosis. In a prospective study involving healthy family members of a patient with CD, elevated levels of this acid were associated with the subsequent development of the disease. This underscores the role of tryptophan metabolites as a potential predictive factor in IBD; however, further research in this area is necessary [150]. Serotonin may also serve as a potential biomarker for differentiating CD activity. In a study by Manzella et al. [151], the concentrations of tryptophan, kynurenine, and serotonin in the serum of patients with CD and UC were measured using LC-MS/MS (liquid chromatography with tandem mass spectrometry). The researchers observed significantly higher plasma serotonin concentrations in patients with active CD compared to those in remission or with treatment-resistant disease. In patients with UC, no significant differences were found between the groups. Wang et al. [127] demonstrated that IBD may be associated with reduced 5-HTP levels and impaired serotonin synthesis in the intestinal mucosa. Discrepancies with studies indicating increased serotonin levels in the blood may be related to differences in the biological material analyzed and the stage of serotonin metabolism being assessed [127,151]. Increased activity of the serotonin pathway may be associated with heightened inflammation within the intestine; however, this issue still requires further clinical research [151]. In the case of indole derivatives, specifically IPA, researchers have shown that its concentration was reduced in the serum of patients with active colitis. For this reason, IPA could potentially be considered a biomarker of intestinal inflammation; however, this hypothesis requires further clinical studies to thoroughly investigate its role as a biomarker [152]. In a study by Zhang et al. [153], the synergistic effect of bacteria, flavonoids, and indole metabolites supported the balance of the gut microbiota, exerted antioxidant effects, and regulated the immune response by reducing pro-inflammatory immune activity and promoting an anti-inflammatory response. Modifying the gut microbiota may also be a promising therapeutic strategy for IBD, which in turn could increase the production of beneficial tryptophan metabolites [9,154]. To summarize the findings to date, tryptophan metabolites may play a significant role in regulating inflammatory processes; however, further clinical studies are necessary to gain a more precise understanding of all mechanisms of action. A more detailed understanding of their impact on, among other things, AhR receptor activity and the functioning of the intestinal barrier could lay the groundwork for the development of new therapeutic strategies for IBD [155]. Future clinical studies should focus on the simultaneous analysis of the kynurenine, serotonin, and indole pathways in patients with IBD. Studies involving large cohorts that consider disease activity, dietary habits, the chosen treatment method, and the composition of the gut microbiota will be of particular importance. It is also necessary to standardize methods for measuring metabolites as potential biomarkers of disease activity and response to therapy [127,132,143,156].

## 5. Clinical Translation

Analysis of the human metabolome reflects the metabolic activity of the gut microbiota. Metabolites appear to be promising diagnostic and prognostic biomarkers. Despite advances in IBD pharmacotherapy, including biologic therapies and small-molecule drugs, a substantial proportion of patients either fail to achieve an initial response or experience a loss of response over time [157]. Because IBD encompasses various disease entities (e.g., CD, UC) with different courses, this poses serious challenges for both clinical treatment and translational research [158]. To enable personalized therapy for a specific patient, a multidimensional, biomarker-based approach is necessary, combining genetic, microbiological, metabolic, and other data [159]. Changes in the levels and ratios of certain biomarkers in patients with IBD are increasingly being identified, creating opportunities for earlier intervention [157].

### 5.1. Potential Diagnostic and Prognostic Biomarkers

Changes in the levels of metabolites derived from the gut microbiome in IBD offer diagnostic and prognostic opportunities for this group of diseases. Three key groups of metabolites—SCFAs, tryptophan metabolites, and bile acid metabolites—form the basis of biomarker research [5]. SCFAs show promising potential as biomarkers in IBD; however, the evidence is mixed and limited due to the heterogeneity of the studies [160]. In one study involving adult patients with IBD, no differences in SCFA levels were observed compared to the control group [161]. Similarly, Ilyés et al. [162] found no differences in SCFA levels between the study patients and a group of healthy individuals. Tews et al. [53] observed subtle differences in SCFA levels in patients with UC; however, the authors themselves acknowledge that SCFAs had no diagnostic value, could not distinguish CD from UC, nor reliably monitor disease activity. Saedi et al. [163] demonstrated that butyric acid levels, in particular, may be reduced in patients with IBD. Gray et al. [164], on the other hand, point out that when using SCFAs as biomarkers for IBD, the metabolite profile should be considered, particularly the ratios between SCFAs and branched-chain fatty acids (BCFAs). Additionally, they report greater sensitivity and specificity for the examined metabolite ratios compared to fecal calprotectin. Meta-analyses evaluating SCFAs as potential biomarkers of IBD activity found that both butyrate and acetate were reduced in patients with IBD, particularly during active phase [17,49]. Methodological heterogeneity, differences in sample size, disease type, and disease activity contribute to these discrepancies; therefore, SCFAs as potential biomarkers in IBD require further, well-designed human studies.

Bile acid (BA) metabolites show potential as diagnostic and prognostic biomarkers in IBD; however, as with SCFAs, their application in clinical practice is limited [156]. Xu et al. [100] observed abnormalities in BA metabolism in patients with IBD, which may result from gut dysbiosis, progressive inflammation, and disturbances in intestinal barrier homeostasis. The authors suggest that BAs may serve as a potential diagnostic marker for IBD; however, given the differences between CD and UC, further research in this area is necessary. Another study also found significant differences in BA levels between active and remission phases of IBD. The BA profile may serve as a potential biomarker for monitoring disease activity or assessing response to treatment [165]. In their study of pediatric patients with CD, Wang et al. [166] observed reduced levels of deoxycholic acid (DCA), lithocholic acid (LCA), and hyodeoxycholic acid (HDCA). Changes in the BA profile may correlate with disease severity, primarily in UC [107]. In IBD, abnormalities in BA metabolism are observed, including the accumulation of primary bile acids and a probable impairment of their bacterial conversion to secondary forms [167]. Liu et al. [168] noted that in patients with CD, higher baseline concentrations of DCA, GDCA, and TDCA predicted a mild disease course over 1.5 years, whereas in patients with UC, higher concentrations of DCA and GDCA and lower concentrations of dehydrocholic acid were associated with a mild disease course over 4 years. Furthermore, high blood levels of 7α-hydroxy-4-cholesten-3-one, indicative of bile acid metabolism disorders, may help identify IBD patients who respond better to fecal microbiota transplantation therapy and have a higher chance of achieving remission [169]. Bile acid profiles show great potential as future diagnostic and prognostic markers in IBD; however, their clinical application requires further validation and standardization of analytical methods [156].

Tryptophan (Trp) metabolites also show great potential as diagnostic biomarkers in IBD. The three tryptophan metabolic pathways—the kynurenine pathway (accounting for approximately 95% of metabolized Trp), the indole pathway (approximately 4–6% of tryptophan), and the serotonin pathway (approximately 1–2% of tryptophan)—exhibit abnormalities in IBD, which may serve as the basis for potential disease biomarkers. In IBD, there is a shift in balance from the protective indole pathway toward the kynurenine pathway, which is associated with enhanced kynurenine pathway activity and alterations in kynurenine and its downstream metabolites involved in the regulation of immune and inflammatory responses. Maaß et al. [170] examined Trp and CRP levels in patients with IBD during induction treatment with various advanced therapies, including TNF-α inhibitors, interleukin inhibitors, JAK inhibitors, sphingosine-1-phosphate receptor modulators, and integrin inhibitors. In their poster presentation, they reported that higher serum tryptophan concentrations were associated with lower CRP levels, with the strongest associations observed in patients receiving interleukin or JAK inhibitors. Karakan et al. [171], also in a poster presentation, show that tryptophan metabolism disorders are closely linked to IBD activity. Additionally, a decrease in tryptophan concentration and an increase in the KYN/TRP ratio may indicate an exacerbation of the inflammatory process. Another study also demonstrated that elevated KYN/TRP ratios were strongly associated with the active phase of the disease. Additionally, they were correlated with fecal calprotectin levels and decreased as patients’ clinical condition improved [172]. Furthermore, analysis of Trp metabolite profiles allows for the identification of certain metabolotypes in patients with IBD. An example is a high concentration of kynurenic acid, which was associated with a milder course of IBD, whereas low levels of kynurenic acid (KYNA) or elevated quinolinic acid (Quin) indicated greater disease activity and a risk of progression [173]. In the study by Michaudel et al. [174], the authors also indicate that specific Trp metabolomic profiles may reflect disease activity and the risk of its progression. Additionally, they describe shifts in the balance between protective (KYNA, xanthurenic acid (XANA)) and potentially pro-inflammatory (QUIN) metabolites of the kynurenine pathway.

The biological matrix should be considered when evaluating metabolite-based biomarkers in IBD. Fecal metabolites primarily reflect local intestinal microbial metabolism, whereas circulating metabolites additionally reflect intestinal absorption and systemic metabolism. Therefore, findings obtained from fecal and blood samples may provide complementary rather than directly interchangeable information [164,165].

Despite these promising results, they should be confirmed in larger, multicenter, and long-term studies. A summary of the potential diagnostic and prognostic value of selected gut microbiota metabolites for IBD is presented in Table 3.

### 5.2. Predictive Biomarkers

The therapeutic approaches evaluated in studies investigating metabolite-based predictors of treatment response in IBD include biologic therapies, small-molecule drugs, and nutritional interventions. Among biologic agents, infliximab targets TNF-α, whereas vedolizumab targets the α4β7 integrin and thereby inhibits gut-selective lymphocyte trafficking. Other advanced therapies discussed in this section include interleukin inhibitors and JAK inhibitors [176,177].

Gut microbiota metabolite profiles may also serve as potential predictors of treatment response in patients with IBD. In a systematic review encompassing 28 studies, Meade et al. [178] noted that patients who responded to treatment exhibited greater activity in butyrate biosynthesis pathways and increased metabolic exchange between bacteria. Furthermore, patients responding to biologic therapy had higher concentrations of secondary bile acids and greater activity of bile acid-metabolizing bacteria. In contrast, Ottria et al. [179] indicate that the current evidence supporting the predictive value of SCFAs with regard to vedolizumab is inconsistent. The potential of SCFAs as biomarkers of treatment response may depend on the therapy used, which requires confirmation in future studies. In BA, a predominance of secondary bile acids is associated with a better response to anti-TNF therapy and anti-cytokine therapy, suggesting that the bile acid profile may serve as a promising predictive biomarker of treatment efficacy in IBD [81]. Furthermore, reduced levels of taurochenodeoxycholic acid and an altered bile acid profile in patients with IBD were associated with a lack of response to infliximab [180]. In an analysis of preliminary data, Reider et al. [181] demonstrated that, in patients treated with vedolizumab, the baseline metabolomic profile was associated with response to therapy. Cai et al. [78] indicate that the profile of secondary bile acids (glycolithocholic acid, GDCA, and ursodeoxycholic acid) could be linked to response to biologic therapy in IBD, suggesting their potential role as predictive biomarkers of treatment response. It has also been observed that reduced abundance of the *bai* operon in the gut microbiome predicted lower levels of secondary bile acids in IBD, which could indirectly serve as a predictor of treatment response [89,182]. In turn, Trp metabolites were identified by Sigall Boneh et al. [183] as a predictive factor for nutritional treatment in pediatric patients with CD. They demonstrated that elevated levels of kynurenine in stool prior to treatment were associated with a failure to achieve remission after 6 weeks of therapy with an exclusion diet combined with partial enteral nutrition (CDED  +  PEN) or exclusive enteral nutrition. Maaß et al. [170] observed that higher serum tryptophan concentrations correlated with a lower CRP levels during treatment with interleukin and JAK inhibitors. Another study found that the normalization of tryptophan metabolism abnormalities following successful treatment with infliximab may reflect a therapeutic response [184].

Several factors should be considered when interpreting the predictive value of metabolite-based biomarkers in IBD. Metabolite profiles may be influenced by the type of therapy, disease activity, dietary intake, and gut microbiota composition. In addition, methodological differences between studies, including the type of biological sample analyzed, timing of sample collection, and analytical methods used, may contribute to variability in the observed predictive performance. These factors should therefore be carefully considered in future studies evaluating metabolites as potential predictors of treatment response in IBD [156,160,185,186].

To date, studies indicate that changes in the metabolite profile and the activity of metabolic pathways may serve as promising tools for predicting treatment response in IBD; however, further validation of these markers is necessary.

## 6. Limitations and Research Gaps

Despite the growing interest in gut microbiota-derived metabolites, their clinical relevance in IBD remains incompletely established. Important gaps concern the reproducibility of reported metabolite patterns and their relationships with specific disease phenotypes and treatment outcomes. Future research should focus on larger, multicenter and longitudinal studies using standardized analytical approaches to determine the clinical utility of these metabolites. Particular attention should be given to identifying metabolite profiles that may support disease monitoring and treatment stratification. Another limitation is the limited specificity of individual microbiota-derived metabolites, as similar alterations may occur in other intestinal diseases. Therefore, single metabolites may not be sufficient for the differential diagnosis of IBD, while multi-metabolite panels may provide greater diagnostic value and should be further investigated.

## 7. Conclusions

Although metabolites derived from the gut microbiota show potential as biomarkers and therapeutic opportunities in IBD, their clinical utility remains insufficiently validated for routine practice. For SCFAs, reduced fecal butyrate and acetate concentrations have been reported in IBD, but the diagnostic and monitoring value of fecal SCFAs remains uncertain. Mechanistic evidence for SCFAs, particularly butyrate, is still derived largely from preclinical and ex vivo studies, while the clinical efficacy of SCFA supplementation remains inconclusive and may be influenced by delivery to the colon and the baseline composition of the gut microbiota. For bile acids, the most consistent alteration is a decrease in the proportions of DCA, LCA, and their derivatives, accompanied by a relative increase in primary and conjugated bile acids, particularly in active UC. Bile acid profiles have also shown potential for predicting treatment response. Tryptophan metabolism is also altered in IBD, with reduced serum tryptophan concentrations and evidence of altered kynurenine pathway activity, including increased IDO activity and an elevated KYN/TRP ratio. Reduced levels of selected microbiota-derived AhR ligands have also been reported. Across these metabolite classes, the current predictive findings cannot yet be directly applied to clinical treatment stratification. Most available prediction studies are observational, and validation in independent external cohorts remains limited. Approaches combining multiple metabolites with microbiota and inflammatory markers appear more promising than approaches based on individual metabolites. Clinical translation will require prospective, longitudinal, multicenter studies using standardized analytical methods, accounting for relevant clinical, dietary, microbiological, and methodological factors, and including validation in independent cohorts.

## Figures and Tables

**Table 1:** Clinical evidence on short-chain fatty acid supplementation in inflammatory bowel disease.

Study	Population	Intervention	Results	Limitations	References
Serrano-Fernández et al. 2025	Systematic review with meta-analysis; 10 included studies; 389 adult patients with active UC reported across studies	Oral sodium butyrate: 600 mg/d or 4 g/d; rectal butyrate: 8–20 mmol/d	Oral 600 mg/d reduced fecal calprotectin and CRP; 4 g/d was associated with clinical, endoscopic, and histological improvement. Rectal administration showed no significant pooled improvement in clinical activity or histology; no adverse effects were reported.	Gut microbiota was not assessed; evidence for oral administration was limited.	[57]
Firoozi et al. 2024	Double-blind RCT; *n* = 36; mild-to-moderate active UC	Sodium butyrate 600 mg/d, 12 weeks	Reduced fecal calprotectin and high-sensitivity CRP and improved sleep quality and quality of life compared with placebo.	Gut microbiota was not assessed; no butyrate measurement in stool samples	[58]
Karłowicz et al. 2025	Multicenter double-blind RCT; *n* = 107 randomized; *n* = 98 per-protocol; mild-to-moderate active UC	Microencapsulated sodium butyrate 600 mg/d, 8 weeks; add-on to stable standard therapy	Per-protocol: clinical remission 31.4% vs. 6.4%; endoscopic improvement 25.5% vs. 4.6%. Among patients achieving clinical remission, an increase in fecal butyrate concentration correlated with improvement on the Mayo scale (*rho* = 0.80 for the overall score; *rho* = 0.87 for the endoscopic score).	Gut microbiota was not assessed; short intervention duration.	[63]
Facchin et al. 2026	Double-blind RCT; *n* = 152 randomized; *n* = 140 analyzed; CD and UC	Microencapsulated sodium butyrate 1200 mg/d, corresponding to 600 mg of active compound, for 90 days	In CD, disease activity and fecal calprotectin improved significantly. Microbiota effects were most pronounced in Enterotype 1, with a greater increase in the *Firmicutes*/*Bacteroidota* ratio, increases in selected SCFA-producing taxa, and reductions in several potentially pathogenic taxa. Corresponding clinical and microbiota effects were less evident in UC.	Small subgroup sizes; requires prospective confirmation with objective endpoints	[64]

Abbreviations: CD, Crohn’s disease; UC, ulcerative colitis; RCT, randomized controlled trial; SCFA, short-chain fatty acids; CRP, C-reactive protein.

**Table 2:** Bile acid profile changes in Crohn’s disease and ulcerative colitis.

Groups	Bile Acid	Crohn’s Disease	Ulcerative Colitis	Potential Clinical Implications	References
Primary bile acids	CA	In 62 patients with CD and 33 healthy control serum concentrations are usually unchanged or variable; the profile depends on the location of the disease and the extent of ileal involvement.	In 32 patients with UC and 23 healthy controls increased concentration and proportion of bile acids in the fecal pool, particularly in active disease.	Fecal CA is increased in UC, whereas serum CA remains unchanged in CD, indicating that its clinical relevance depends on the biological material analyzed.	[99,101,107]
	CDCA	Results are variable; levels may be reduced in the blood circulation in cases of intestinal malabsorption.	Fecal CDCA concentrations showed a non-significant upward trend, while its conjugated forms, GCDCA and TCDCA, were elevated in active UC.	CDCA findings are inconsistent across studies and sample types: serum CDCA may be reduced in IBD, while fecal CDCA itself is not significantly increased in UC, although its conjugated forms GCDCA and TCDCA are elevated.	[99,100]
Primary conjugated bile acids	GCA	In 74 patients with CD and 71 controls, increased proportion of bile acids in the plasma pool.	In cohorts including 269 patients with IBD this increase is observed in some individuals, particularly in cases of active inflammation.	Increased GCA has been reported in circulating bile acid profiles in CD/IBD, but its individual diagnostic performance is limited.	[98,100]
	TCA	Concentrations vary depending on the biological material and the disease phenotype.	In 32 patients with UC and 23 controls, and in a second cohort including 24 UC patients elevated levels in stool, especially in more severe UC.	TCA alterations are sample- and disease-dependent, with reduced plasma proportions in CD but increased fecal concentrations in UC.	[98,101,107,109]
	GCDCA	Reduced plasma levels and reduced ability of the entire BA profile to activate FXR.	Elevated fecal concentrations, associated with active inflammation.	GCDCA shows opposite patterns in blood and stool, with reduced plasma proportions in CD and increased fecal concentrations in UC.	[98,101]
	TCDCA	No definite direction of change.	Elevated fecal concentration in active UC.	Fecal TCDCA is increased in UC and contributes to the altered primary conjugated bile acid profile observed in these patients.	[101]
Secondary bile acids	DCA	Reduced levels, particularly in active disease; a lower DCA/(DCA + CA) ratio correlates with higher CD activity.	Reduced levels and fecal excretion; inversely correlated with markers of inflammation.	Reduced DCA is associated with active UC, particularly diarrhea and inflammatory activity, and may reflect altered microbial bile acid metabolism.	[99,101,107]
	LCA	Reduced plasma levels; the extent of the changes depends on the location and activity of the disease.	Reduced fecal concentrations, particularly during active inflammation.	Reduced fecal LCA is associated with active UC, particularly diarrhea and higher inflammatory activity, indicating altered secondary bile acid metabolism.	[99,101,107]
Secondary conjugated bile acids	GDCA	Lower concentration in active CD than in remission; inversely correlated with the CDAI.	Reduced fecal concentration, particularly in patients with severe symptoms.	GDCA shows opposite patterns depending on the biological material, with an increased plasma proportion in CD and reduced fecal concentrations in UC.	[98,101,107]
	GLCA	Data specific to CD are limited.	Reduced fecal concentrations and an inverse correlation with calprotectin.	Reduced GLCA levels are observed in both serum IBD profiles and fecal samples from UC patients, indicating a consistent decrease in this secondary bile acid derivative.	[100,101,107]
	TLCA	This reduction has been described primarily in analyses of the overall IBD population.	Reduced concentrations in stool and an inverse correlation with CRP.	TLCA is reduced in serum IBD profiles and in fecal samples from UC patients and is inversely associated with pro-inflammatory cytokines.	[100,101]
Other secondary BA	HDCA/GHDCA	In a cohort of 38 CD patients, 24 UC patients and 17 controls, GHDCA is usually reduced; HDCA may be higher than in UC.	Reduced concentrations, especially when calprotectin is high.	Reduced HDCA is associated with higher inflammatory activity in UC, while relative GHDCA levels show potential for distinguishing UC from CD.	[107]
Microbiological derivatives	3-oxo-DCA, 3-oxo-LCA, isoLCA, allo-LCA, isoallo-LCA	Reduced, although data distinguishing CD phenotypes are limited.	In 95 treatment-naïve pediatric patients with UC, particularly reduced in active and severe UC.	Reduced levels of microbiota-derived bile acid metabolites are associated with active and severe UC, reflecting altered microbial bile acid metabolism.	[86,109]
Aggregate Indicators	Ratio of Secondary to Primary BA	Reduced in active CD, particularly in serum.	Markedly reduced in stool, especially in active UC.	A reduced secondary-to-primary BA ratio is associated with active CD and is significantly decreased in fecal samples from UC patients, reflecting altered bile acid metabolism in IBD.	[99,107]

Abbreviations: BA—bile acids; CA—cholic acid; CDCA—chenodeoxycholic acid; DCA—deoxycholic acid; LCA—lithocholic acid; GCA—glycine-conjugated cholic acid; TCA—taurocholic acid; GCDCA—glycine-conjugated chenodeoxycholic acid; TCDCA—tauro-conjugated chenodeoxycholic acid; GDCA—glycine-conjugated deoxycholic acid; GLCA—glycine-conjugated lithocholic acid; TLCA—tauro-conjugated lithocholic acid; HDCA—hydrodeoxycholic acid; GHDCA—glycine-conjugated hydrodeoxycholic acid; 3-oxo-DCA—3-oxo-deoxycholic acid; 3-oxo-LCA—3-oxo-lithocholic acid; isoLCA—iso-lithocholic acid; allo-LCA—allo-lithocholic acid; isoallo-LCA—isoallo-lithocholic acid; FXR—FXR nuclear receptor (farnesoid X receptor); CD—Crohn’s disease; UC—ulcerative colitis; IBD—inflammatory bowel disease; CDAI—Crohn’s Disease Activity Index; CRP—C-reactive protein.

**Table 3:** Diagnostic and prognostic value of gut microbiota metabolites in inflammatory bowel disease.

Metabolite	Study Population	Sample	Direction of Change in Inflammatory Bowel Disease	Potential Application	References
Short chain fatty acids (SCFAs)	18 patients with Crohn’s disease (CD), 29 patients with ulcerative colitis (UC), and 31 healthy controls (HC)	Serum	No significant differences in total serum SCFA concentrations between CD, UC, and HC	Limited diagnostic value, further validation is required.	[161]
SCFAs	40 patients with IBD (UC and CD), 20 individuals in the control group	Feces (stool)	Decreased butyric acid levels in patients with UC and CD. Decreased acetic and propionic acid levels were observed in patients with UC.	Potential indicator of altered gut microbial metabolism and SCFA production.	[163]
SCFAs	29 patients with IBD, 30 individuals in the control group	Serum	No significant differences in serum total SCFA concentrations between the IBD and control groups.	Further research is required to determine their clinical relevance.	[162]
SCFAs	64 patients with IBD, 20 patients with PSC-IBD, and 17 healthy controls	Feces (stool)	Fecal acetate, propionate, and butyrate levels were similar in patients with IBD and healthy controls, whereas isobutyrate was decreased in IBD. SCFAs did not discriminate between CD and UC.	No confirmed diagnostic value for IBD diagnosis or monitoring disease severity.	[53]
SCFAs	Meta-analysis (9 studies) Patients with IBD	Feces (stool)	↓ butyrate (especially in active IBD); ↓ acetate; ↓ propionate.	Potential marker of altered microbial metabolism in IBD, further standardization and validation are required.	[17]
SCFAs	Meta-analysis (12 studies) Patients with IBD	Feces (stool)	Overall: ↓ acetate, ↓ propionate, ↓ butyrate, ↓ valerate, and ↑ lactate. SCFA alterations differed between CD and UC and according to disease activity.	The SCFA profile may reflect alterations in microbial metabolism and differences in IBD phenotype and disease activity.	[49]
Bile acid (BA)	269 patients with IBD, 200 healthy controls (HC), 174 individuals with other intestinal diseases (OID)	Serum	↓ CDCA, DCA, GDCA, TDCA, LCA, GLCA, and TLCA; ↑ primary/secondary BA ratio (IBD vs HC).	Potential diagnostic biomarker for distinguishing IBD from healthy controls.	[100]
BA	Pediatric IBD: Cohort A: 37 active IBD cases, 33 in remission, 11 healthy controls; Cohort B: 59 active IBD cases, 80 in remission, 61 other inflammatory bowel diseases, 38 healthy controls; Cohort C: 17 active IBD cases, 31 in remission	Feces (stool) and serum	Active IBD: ↑ primary BAs (including TCA and GCA); ↓ secondary BAs (including DCA, UDCA, and GUDCA).	Potential biomarker for assessing disease activity in pediatric IBD.	[165]
BA	62 patients with IBD, 17 individuals in the control group	Feces (stool)	UC: ↓ DCA, LCA, and total secondary BAs. Lower secondary BA levels were associated with ↑ CRP and ↑ fecal calprotectin.	Potential biomarker of disease activity and severity in UC.	[107]
BA	Discovery cohort: 155 patients (68 CD, 53 UC, 34 HC); validation cohort: 65 patients (20 CD, 23 UC, 22 HC)	Feces (stool)	↑ primary BAs (CA and CDCA); LCA and DCA showed decreasing trends in CD.	Potential marker of altered microbiota–bile acid metabolism in IBD.	[167]
BA	20 healthy children, 29 children with CD	Feces (stool)	↑ primary BA, ↓ secondary BA, ↓ secondary/primary BA ratio.	Potential marker of altered gut microbial bile acid metabolism in pediatric CD.	[166]
Tryptophan (Trp)	25 patients with IBD (15 with UC, 10 with CD)	Serum	A negative correlation between TRP and CRP. Higher TRP levels are associated with lower inflammatory activity.	Potential marker of treatment response, particularly in UC patients receiving interleukin or JAK inhibitors.	[170]
Trp	115 patients with IBD (77 with UC, 38 with CD)	Serum	Active IBD: ↓ Trp, ↓ KYNA, ↓ picolinic acid; ↑ KYN/Trp ratio	Potential biomarker of IBD disease activity.	[171]
Trp	55 patients with IBD (27 with UC, 28 with CD), 50 healthy individuals	Serum	↓ serum TRP; ↓ TRP/KYN; IBD compared with the control group	Potential marker of altered kynurenine pathway metabolism in IBD.	[175]
Trp	55 HC, 55 UC (35 active, 20 in remission), 30 CD (20 active, 10 in remission)	Plasma	↑ KYN/TRP in active IBD compared to remission and healthy individuals. KYN/TRP correlated with fecal calprotectin.	Potential biomarker for assessing and monitoring IBD disease activity.	[172]

Abbreviations: SCFA—short-chain fatty acids, CD—Crohn’s disease, UC—ulcerative colitis, HC—healthy controls, IBD—inflammatory bowel disease, PSC-IBD—primary sclerosing cholangitis-associated IBD, BA—bile acids, OID—other intestinal diseases, DCA—deoxycholic acid, LCA—lithocholic acid, GDCA—glycodeoxycholic acid, TDCA—taurodeoxycholic acid, GLCA—glycolithocholic acid, TCA—taurocholic acid, GCA—glycocholic acid, UDCA—ursodeoxycholic acid, CRP—C-reactive protein, Trp—tryptophan, KYN—kynurenine, KYNA—kynurenic acid, KYN/TRP—kynurenine-to-tryptophan ratio. ↑, increased; ↓, decreased.

## Data Availability

No new data were created or analyzed in this study. Data sharing is not applicable to this article.

## Abbreviations

The following abbreviations are used in this manuscript:

IBD	inflammatory bowel diseases
CD	Crohn’s disease
UC	ulcerative colitis
SCFAs	short-chain fatty acids
BAs	bile acids
CRP	C-reactive protein
ESR	erythrocyte sedimentation rate
AhR	aryl hydrocarbon receptor
HCAR2	hydroxycarboxylic acid receptor 2
Treg	regulatory T cell
Tr1	type 1 regulatory T cells
moDC	monocyte-derived dendritic cells
HDAC	histone deacetylases
HIF	hypoxia-inducible factor
MSB	microencapsulated sodium butyrate
AGA	American Gastroenterological Association
CYP	cytochrome P450
CA	cholic acid
CDCA	chenodeoxycholic acid
DCA	deoxycholic acid
LCA	lithocholic acid
FXR	farnesoid X receptor
TGR5	G protein-coupled bile acid receptor 1
FGF19	fibroblast growth factor 19
TJPs	tight junction proteins
ILC3	group 3 innate lymphoid cells
GDCA	glycodeoxycholic acid
CDAI	Crohn’s Disease Activity Index
FMT	fecal microbiota transplantation
TRP	tryptophan
KP	kynurenine pathway
TDO2	tryptophan 2,3-dioxygenase 2
AA	anthranilic acid
KA	kynurenic acid
3-HK	3-hydroxykynurenine
3-HAA	3-hydroxyanthranilic acid
QA	quinolinic acid
5-HT	5-hydroxytryptamine
TPH1	tryptophan hydroxylase 1
AADC	aromatic amino acid decarboxylase
5-HTP	5-hydroxytryptophan
AANAT	arylalkylamine N-acetyltransferase
HIOMT	hydroxyindole-O-methyltransferase
IPYA	indole-3-pyruvate
ArAT	aromatic amino acid aminotransferase
ILA	indole-3-lactic acid
fldH	phenyllactate dehydrogenase
IdhA	D-2-hydroxyacid dehydrogenase
IA	indoleacrylic acid
IPA	indole-3-propionic acid
IAAld	indole-3-acetaldehyde
IAA	indoleacetic acid
IAld	indole-3-aldehyde
ICA	indole-3-carboxylic acid
IAM	indole-3-acetamide
HPLC	high-performance liquid chromatography
TJ	tight junction
AJ	adherens junction
AJC	apical junction complex
BCFAs	branched-chain fatty acids
